# Pyogenic tenosynovitis of the wrist due to *Corynebacterium striatum* in a patient with dermatomyositis: A case report

**DOI:** 10.1097/MD.0000000000018761

**Published:** 2020-01-17

**Authors:** Yohei Kawaguchi, Hideki Okamoto, Kojiro Endo, Hidetoshi Iwata, Yuji Joyo, Masahiro Nozaki, Shinya Tamechika, Yuko Waguri-Nagaya, Hideki Murakami

**Affiliations:** a Department of Orthopedic Surgery; b Department of Glial Cell Biology; c Department of Respiratory Medicine, Allergy and Clinical Immunology, Nagoya City University Graduate School of Medical Sciences, Mizuho-Ku, Nagoya, Japan.

**Keywords:** corticosteroid injection, Corynebacterium striatum, dermatomyositis, immunosuppressants, pyogenic tenosynovitis

## Abstract

**Introduction:**

*Corynebacterium striatum* is common contaminant in clinical specimens. Here, we report a rare case of pyogenic tenosynovitis of the wrist caused by *C striatum* in a dermatomyositis patient taking oral immunosuppressants.

**Patient concerns:**

A 67-year-old Japanese woman with dermatomyositis had a history of multiple intraarticular injections of corticosteroids to the right wrist joint for the treatment of osteoarthritis. She was admitted to our hospital with a painful lump on the right dorsal wrist lasting for three months. MRI revealed cellulitis of the dorsum of the right wrist and hand and fluid collection in the extensor tendon sheath. *C striatum* was detected in the cultures of three samples of synovial fluid taken from the dorsal hand.

**Diagnosis:**

Pyogenic tenosynovitis of the wrist due to *C striatum*.

**Interventions:**

The infection was successfully controlled with synovectomy and adjuvant antibiotic therapy.

**Outcomes:**

There has been no sign of recurrence for 12-months after the surgical treatment.

**Lessons:**

This is the first reported case of pyogenic tenosynovitis due to *C striatum* in a patient with dermatomyositis. Clinicians should be aware that patients undergoing immunosuppressive therapy have a risk of *C striatum* infection.

## 1 Introduction

*Corynebacterium* species are normal human flora that are common contaminants in clinical specimens. Except for *Corynebacterium diphtheriae*, all *Corynebacterium* spp. are ubiquitous skin commensals.^[1]^ However, some reports have highlighted the importance of *C striatum* as causes of catheter-related bloodstream infections and various other infections, such as arthritis and endocarditis, in immunosuppressed patients.^[2–5]^ Here, we report a rare case of pyogenic tenosynovitis of the wrist caused by *C striatum* in a patient with dermatomyositis who was taking oral immunosuppressants. The infection was successfully controlled with synovectomy and adjuvant antibiotic therapy.

## 2 Case presentation

**Figure 1:**
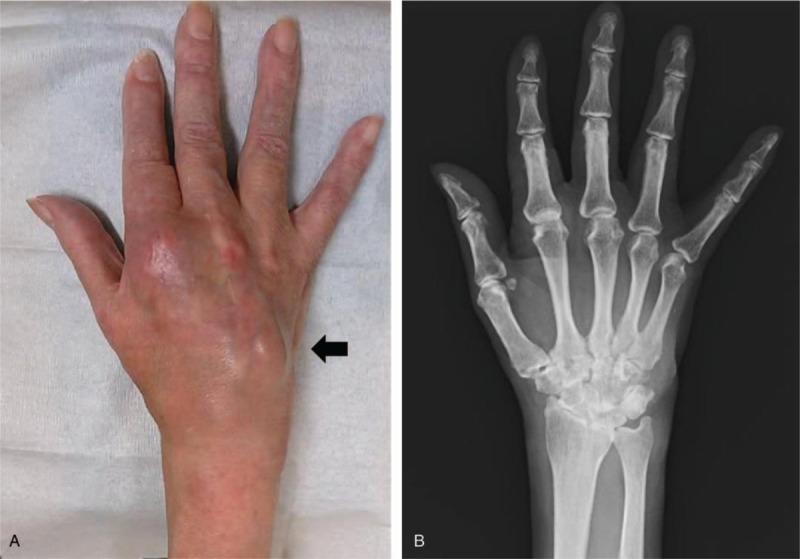
Preoperative appearance and plain radiograph of the right wrist. (A) Lump on the dorsal area of the right wrist and hand (black arrow). (B) Plain radiograph of the right wrist reveals sclerotic changes of the carpal bones and narrowing of the radiocarpal joint space.

**Figure 2:**
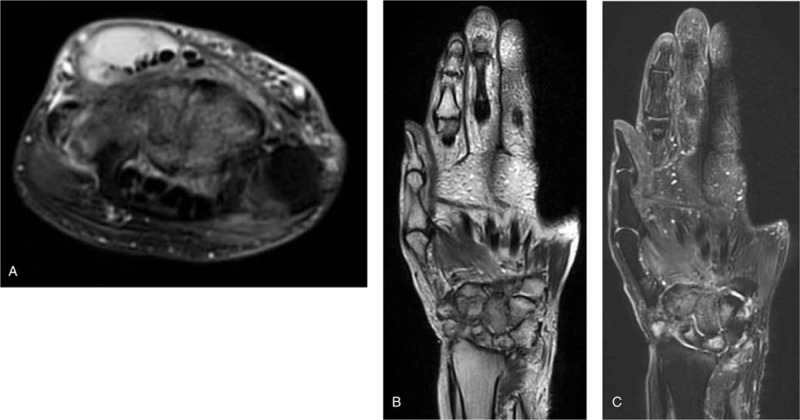
Preoperative MRI of the right wrist. (A) An MRI T2 weighted contrast transverse image reveals diffuse subcutaneous edema in the dorsum of the wrist and fluid collection in the extensor tendon sheath. Coronal image demonstrates the bone marrow edema with low signal intensity in the T1 WI (B) and high signal intensity in the T2 WI (C) in the bone marrow of the carpal bones. MRI = magnetic resonance imaging, WI = weighted image.

**Figure 3:**
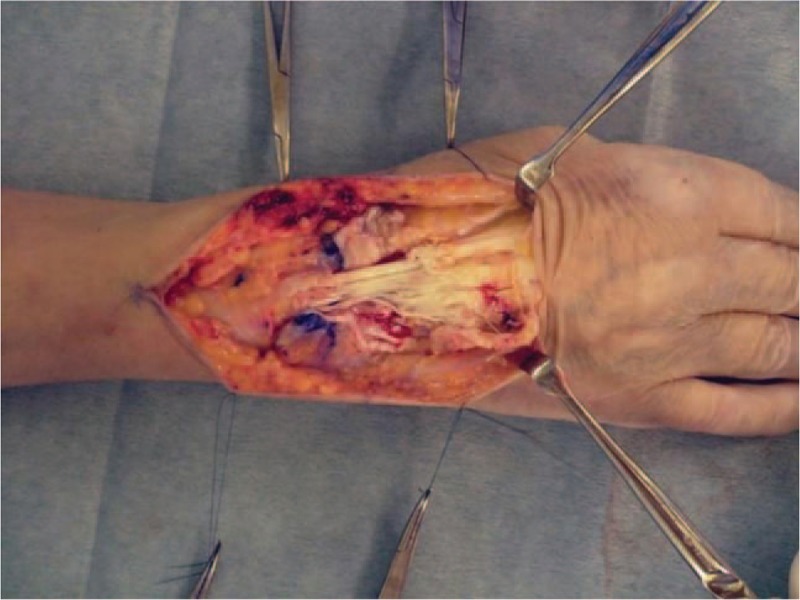
Intraoperative findings. Massive synovitis along the extensor tendons and complete rupture of the extensor tendons of the ring and little fingers.

**Figure 4:**
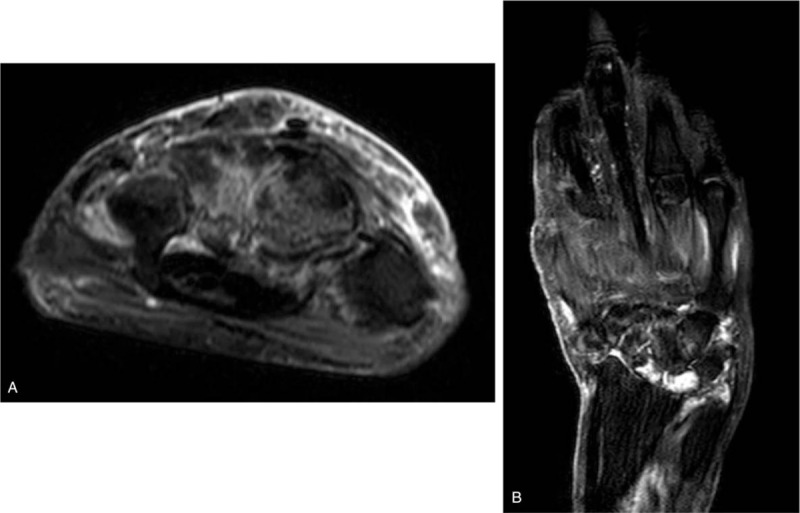
T2 weighted magnetic resonance imaging images at the 6-month follow-up. (A) Synovitis and fluid collection around the extensor tendons disappeared. (B) Signal intensity in the bone marrow of the carpal bones was improved.

A 67-year-old Japanese woman who had had dermatomyositis for 4 years was diagnosed with a typical rash (heliotrope rash, Gottron's sign), symmetric proximal muscle weakness, elevated serum skeletal muscle enzymes, and interstitial lung disease. She had a past history of pleurisy caused by *Mycobacterium abscessus* and mycotic pneumonia. Her dermatomyositis was controlled with prednisolone 5 mg/day and azathioprine 75 mg/day for 3 years. The patient had received clarithromycin (CAM) (400 mg/day) for the treatment of an *M abscessus* infection and itraconazole (100 mg/day) for the treatment of mycotic pneumonia for 1 year, in addition to trimethoprim/sulfamethoxazole (160/800 mg twice weekly) as a prophylaxis for opportunistic infections for 4 years. She had a history of multiple injections of corticosteroids into the right wrist for arthralgia due to osteoarthritis for 6 months. Prior to admission, the patient had a 3-month history of a painful lump on the dorsal area of the right wrist, that had gradually increased in size and finally measured approximately 3 × 5 cm (Fig. 1A). The range of motion of the right radiocarpal joint was limited by pain. The patient was not able to extend the right ring and little fingers. *C striatum* was detected in 3 samples of synovial fluid aspirated from the lump. This was resistant to penicillin and ceftriaxone but sensitive to imipenem, amikacin, vancomycin and rifampicin. Blood biochemistry showed slightly elevated C-reactive protein levels (CRP: 0.47 mg/dl) and a normal white blood cell count (WBC: 7200 /mm^3^) (Table 1). Blood cultures showed no evidence of bacteremia. Plain radiographs of the right wrist revealed narrowing of the radiocarpal and midcarpal joints accompanied by sclerotic changes of the carpal bones due to osteoarthritis (Fig. 1B). Magnetic resonance imaging (MRI) revealed cellulitis of the dorsum of the right wrist and hand and fluid collection in the extensor tendon sheath (Fig. 2A). Additionally, MRI demonstrated bone marrow edema with low signal intensity on T1 weighted-images (WI) and high signal intensity on T2 WI in the carpal bones (Fig. 2B and C). Plain radiographs of the wrist revealed no osteolytic lesion of the carpal bones, and MRI demonstrated fluid within the fourth extensor compartment tendon sheath surrounding the tendons, not in the joint. She was diagnosed as pyogenic extensor tenosynovitis of the wrist due to *C striatum*. Therefore, synovectomy was performed through a dorsal approach. The intraoperative findings revealed massive synovitis along the extensor tendons, with complete rupture of the extensor tendons of the ring and little fingers (Fig. 3). However, there was no intraarticular synovitis. Cultures of synovial tissue was negative, because of intraoperative use of antibiotics. Cultures for tubercle bacillus and polymerase chain reaction for atypical mycobacteriosis were all negative. Histopathology showed synovial tissue with diffuse acute inflammatory infiltrate and the deposition of fibrinous material on the synovial surface, without granulomatous changes. Multidrug chemotherapy including imipenem/cilastatin, daptomycin, and amikacin were initiated. These antibiotics were continued for 2 weeks postoperatively, followed by oral rifampicin 450 mg/day for 3 months. After the 6-month follow-up, MRI showed improved signal intensity in the bone marrow of the carpal bone and extinction of the fluid around the extensor tendons (Fig. 4). There has been no sign of recurrence for 12-months after the surgical treatment. Because the patient feels no disability in daily activities, she does not wish to undergo reconstructive surgery of the extensor tendons at this time.

**Table 1:**
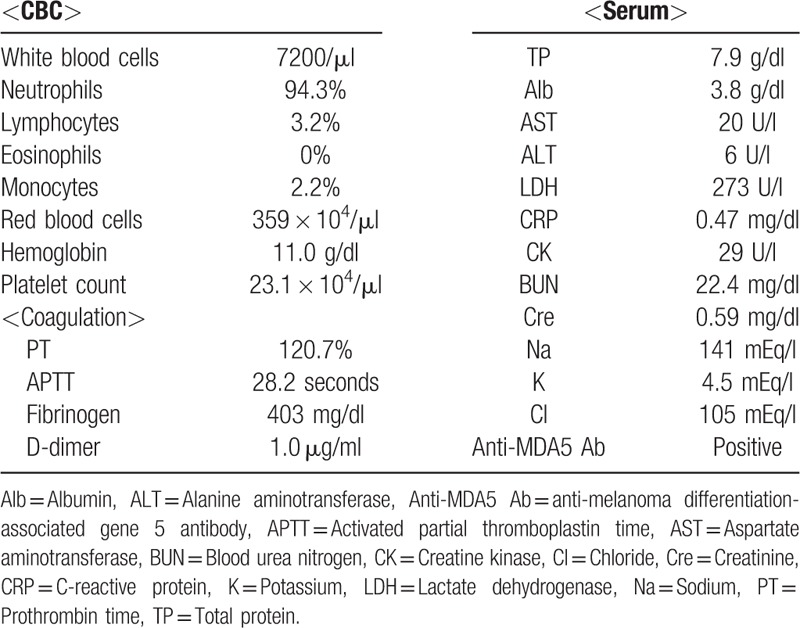
Laboratory findings on admission.

## 3 Discussion

Pyogenic tenosynovitis frequently occurs subsequent to a puncture wound. Therefore, skin flora such as *Staphylococcus aureus* and *Streptococcus species* are found to be the causative organism in most cases. Atypical organisms including gram-negative rods and mixed flora have been found in immunocompromised patients.^[6]^

We presented a case of pyogenic tenosynovitis of the wrist due to *C striatum* in a patient with dermatomyositis who was taking oral immunosuppressants. There have been few reports of pyogenic arthritis and osteomyelitis due to *C striatum*.^[4,7,8]^ To the best of our knowledge, no case of pyogenic tenosynovitis of wrist due to *C striatum* has been reported.

*Corynebacterium* species are opportunistic human pathogens that are distributed in the skin and mucous membranes of asymptomatic individuals. These organisms are often considered contaminants when isolated in culture because of their low virulence.^[9]^ However, when isolated repeatedly in pure growth from a normally sterile body site, such as the synovial fluid or blood, in a clinical context consistent with infection, they should be considered clinically relevant, and identification at the species level and antimicrobial susceptibility testing are recommended.^[9]^ In our case, the first bacterial culture of the fluid was thought to indicate skin contamination; however, *C striatum* was repeatedly detected in the cultures of aspirated material. Therefore, we considered *C striatum* the pathogenic microorganism.

Corticosteroids results in decreased fibrosis and reduced vascular proliferation in peritendinous tissues, along with anti-inflammatory effects that include decreased leukocyte migration and phagocytosis.^[10]^ Saglam et al reported an intratendinous septic abscess of the Achilles tendon after multiple local steroid injections and considered that the abovementioned effect may increase the possibility of infection at the site of local corticosteroid injection.^[11]^ Even in our case, this effect may have been the basis for the infection.

Previously reported risk factors for *C striatum* infection include a history of collagen disease, diabetes mellitus and immunosuppressants therapy.^[4,7,12,13]^ In this case, the patient received prednisolone and an immunosuppressant for approximately 3 years for the treatment of dermatomyositis. Additionally, we concluded that the main cause of the infection was the iatrogenic inoculation of *C striatum* from the bacterial flora of the skin into the extensor tendon sheath.

Treatment of *C striatum* infection includes initiation of appropriate antibiotic therapy and involve possible removal of infectious lesion to ensure complete eradication of the nidus of infection. There is growing concern over resistance of *C striatum* to penicillins, and cephalosporines,^[14]^ as seen in our case, even though previously reported susceptibility to penicillins.^[15]^ Repeated isolation of *C striatum* from a sterile body site should prompt early laboratory request for drug-susceptibility testing. Susceptibility testing is not routinely performed otherwise by most laboratories as *C striatum* is considered a contaminant in most clinical situations. Early detection and appropriate treatment of this infection is important especially in immunosuppressed patients and likely to lead to improved clinical outcomes.

In summary, we reported the case of a patient with pyogenic tenosynovitis due to *C striatum* with dermatomyositis who was treated with the immunosuppressant azathioprine. Surgical radical debridement and multidrug chemotherapy were useful. Meticulous attention should be paid to aseptic techniques whenever local injections of corticosteroids are administered to patients taking immunosuppressants, and patients should be closely observed during the postinjection period.

## Author contributions

**Conceptualization:** Yohei Kawaguchi.

**Data curation:** Yohei Kawaguchi.

**Investigation:** Yohei Kawaguchi, Hideki Okamoto, Kojiro Endo, Hidetoshi Iwata, Yuji Joyo, Masahiro Nozaki, Shinya Tamechika.

**Supervision:** Yuko Waguri-Nagaya, Hideki Murakami.

**Writing – original draft:** Yohei Kawaguchi.

**Writing – review & editing:** Yohei Kawaguchi, Yuko Waguri-Nagaya.
